# A Case of Paget’s Disease of the Breast without a Nipple Lesion

**DOI:** 10.70352/scrj.cr.25-0730

**Published:** 2026-04-10

**Authors:** Yukari Koga, Kyoko Eto, Masumi Tanaka, Yoshiko Masuda, Yasuteru Yoshinaga, Toshihiko Sato, Mikiko Aoki

**Affiliations:** 1 Department of General Thoracic, Breast, and Pediatric Surgery, Fukuoka University Hospital, Fukuoka, Fukuoka, Japan; 2 Department of Pathology, Fukuoka University School of Medicine, Fukuoka University Hospital, Fukuoka, Fukuoka, Japan

**Keywords:** Paget’s disease of the breast, areolar lesion, without nipple involvement, delayed diagnosis, ductal carcinoma in situ

## Abstract

**INTRODUCTION:**

Paget’s disease of the breast is generally associated with breast cancer involving the epithelium of the nipple and frequently extends into the areola or surrounding epidermis. Although it classically presents as a nipple lesion, recent diagnostic frameworks recognize that Paget’s disease may rarely occur without overt nipple involvement. We report a rare case of Paget’s disease of the breast without a nipple lesion.

**CASE PRESENTATION:**

The patient was a 73-year-old woman who was referred to the dermatology department at our hospital for investigation of a ring-shaped area of erythema on her right areola that had been treated with topical steroids for a month at a local dermatology clinic with no improvement. A skin biopsy led to a diagnosis of Paget’s disease of the breast, and she was referred to our department. Mammography and ultrasonography did not show any intramammary lesions, while MRI suggested possible limited ductal involvement near the nipple. However, the exact extent of the disease could not be reliably delineated preoperatively. A right mastectomy was performed. Postoperative pathology confirmed Paget cells in the erythematous area and ductal carcinoma in situ within the breast, without demonstrable histological continuity between the two lesions.

**CONCLUSIONS:**

When a patient presents with a refractory lesion in the areolar region, even in the absence of nipple involvement, the possibility of Paget’s disease of the breast should be considered, and a skin biopsy should be performed for appropriate diagnosis.

## Abbreviations

DCIS
: ductal carcinoma in situ
SNB
: sentinel lymph node biopsy

## INTRODUCTION

Paget’s disease of the breast is a rare condition, accounting for 0.2% of all breast cancers.^1)^ It is a breast cancer with adenocarcinoma components visible within the epithelium of the nipple, often accompanied by pagetoid spread into the areolar region and surrounding epidermis, and frequently presents with intramammary lesions.^2)^ Paget’s disease of the breast typically presents with erythema, erosion, or eczema-like changes of the nipple. However, accumulating evidence indicates that Paget’s disease of the breast may, in rare instances, present without overt nipple involvement, manifesting solely as areolar or periareolar skin lesions. We herein report a rare case of Paget’s disease of the breast discovered solely on the basis of areolar erythema, without any nipple lesions, such as erosion or discharge.

## CASE PRESENTATION

A 73-year-old Japanese woman noticed an annular area of erythema in part of the right areola 2 months before her initial consultation. She visited a local breast surgery clinic, where ultrasonography and mammography did not reveal any lesions. Considering the possibility of a skin disease, she was referred to a local dermatology clinic. Improvement was minimal after 1 month of topical steroid therapy. A biopsy was deemed necessary, and she was referred to our hospital’s dermatology department. A skin biopsy confirmed a diagnosis of Paget’s disease of the breast, and she was referred to our department for further evaluation and treatment.

**Fig. 1:**
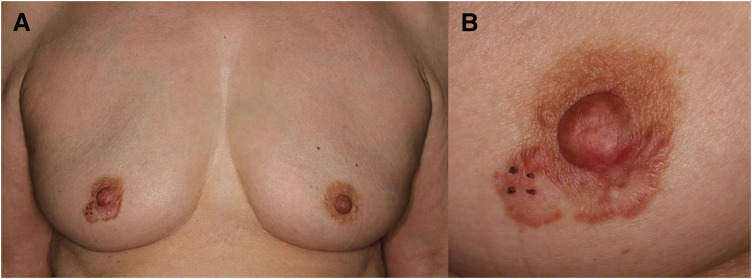
Findings on physical examination. (**A**) A 2-cm annular area of erythema was noted on the right areola. No nipple erosion or nipple discharge was observed. No obvious mass was palpable within the breast. (**B**) A 4-mm skin biopsy was performed in the marked area.

Her past medical history included dyslipidemia, but there was nothing significant in her family history. Physical examination revealed an annular erythematous rash with a diameter of 2 cm on the right areola. No nipple erosion or discharge was observed. No palpable mass was detected in the breast (**Fig. 1**). There were no significant findings in the blood tests.

**Fig. 2:**
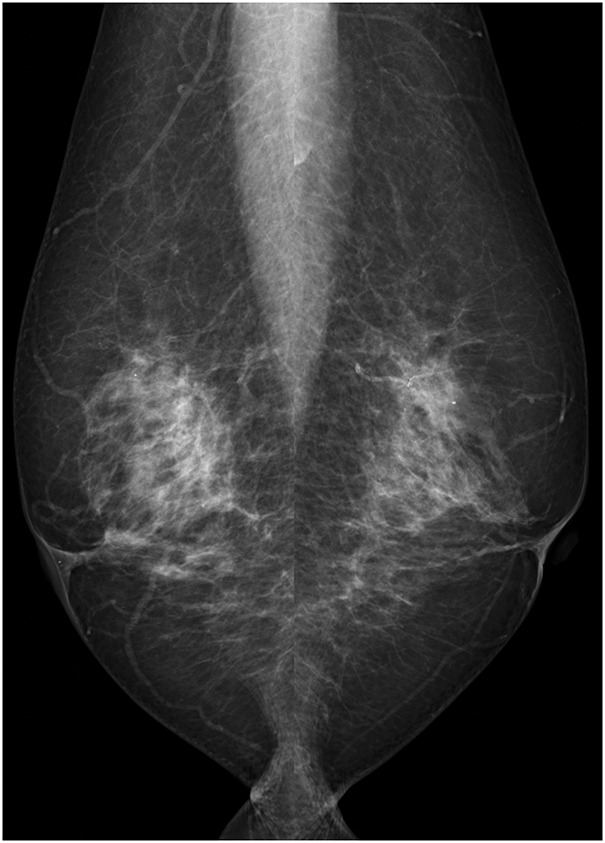
Mammogram showing no evidence of malignancy.

**Fig. 3:**
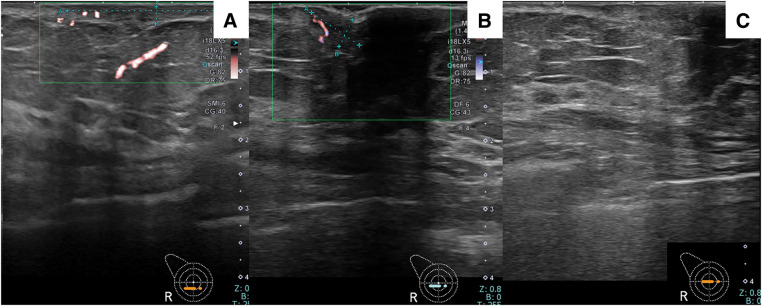
Findings on ultrasonography. (**A**) Erythematous area showing skin thickening. (**B**) An 8.4 × 4.3-mm hypoechoic area with blood flow adjacent to the outer side of the nipple. (**C**) Only scattered 2–3-mm hypoechoic areas were present within the breast. No significant malignant findings were observed.

**Fig. 4:**
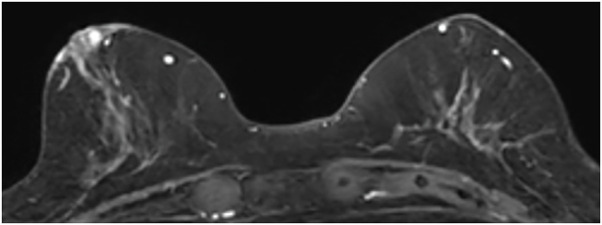
Findings on contrast-enhanced MRI. A 2.5-mm nodule was noted slightly medial to the right nipple. This nodule demonstrated a fast-persistent pattern on contrast enhancement, suggesting possible intraepithelial spread of Paget’s disease into the nipple. Periductal skin thickening was also interpreted as tumor infiltration. Within the breast, small nodules with contrast enhancement were noted within a 2-cm range from the nipple, suggesting ductal spread. No obvious lymph node enlargement was observed.

Mammography revealed scattered mammary glands but no calcifications or masses suggestive of malignancy (**Fig. 2**). Ultrasonography revealed skin thickening in the erythematous area. An 8.4 × 4.3-mm hypoechoic area with blood flow was noted adjacent to the outer side of the nipple. Scattered hypoechoic areas with a diameter of 2–3 mm were present within the breast, with no significant malignant findings (**Fig. 3**). Contrast-enhanced MRI showed a small nodule measuring 2.5 mm in diameter slightly medial to and directly beneath the right nipple. The contrast enhancement pattern was fast-persistent, suggesting possible intraepithelial spread into the nipple. Periductal skin thickening was also considered to indicate tumor infiltration. Within the breast, small enhancing nodules were seen within a range of 2 cm from the nipple, suggesting ductal spread. No obvious lymph node enlargement was observed (**Fig. 4**). Plain CT scans revealed skin thickening in the areolar region. No mass shadow was seen within the breast, and there were no findings suggestive of distant metastasis. The differential diagnosis included Bowen’s disease, Paget’s disease of the breast, and chronic eczema.

**Fig. 5:**
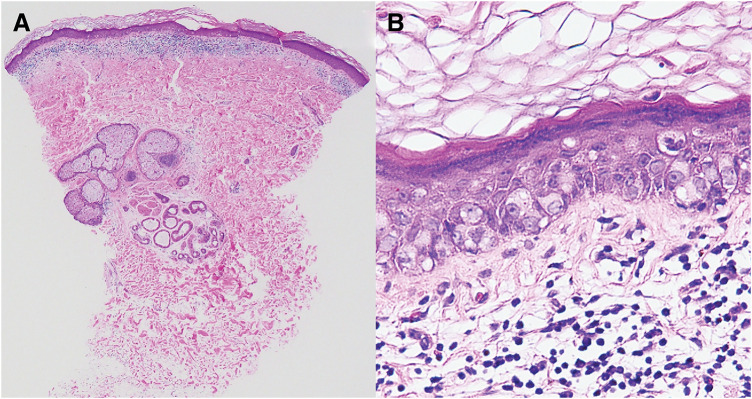
Findings in a skin biopsy. (**A**) Low-magnification image with H&E staining. A proliferation of atypical epithelial cells was noted within the epidermis of the erythematous area; no lesions continuous with the breast tissue were observed. (**B**) High-magnification image with H&E staining. Paget cells with pale cytoplasm were confirmed within the areolar epidermis. H&E, hematoxylin and eosin

A skin biopsy showed proliferation of atypical epithelial cells within the epidermis of the erythematous area. Paget cells with pale cytoplasm were confirmed within the areolar epidermis. No contiguous lesions were seen within the breast (**Fig. 5**).

**Fig. 6:**
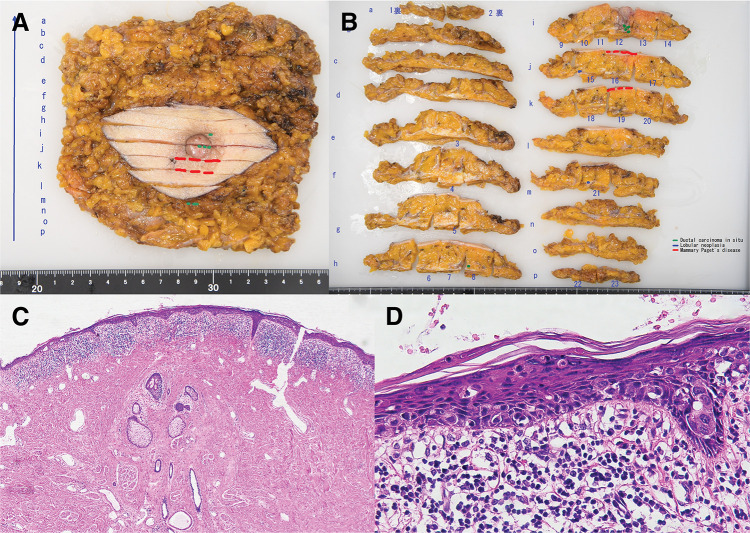
Results of pathological examination. (**A**, **B**) Gross image after formalin fixation. Green indicates DCIS, blue indicates lobular neoplasia, and red indicates the Paget lesion. DCIS was identified immediately beneath the nipple, with lobular neoplasia located deeper and laterally. (**C**) Low-magnification image with H&E staining. Continuity between the skin lesion and the breast lesion could not be confirmed. (**D**) High-magnification image with H&E staining. Scattered atypical (Paget) cells containing mucus were observed within the thickened epidermis of the erythematous area of the areola. DCIS, ductal carcinoma in situ; H&E, hematoxylin and eosin

The diagnosis was Paget’s disease of the right breast, cTisN0M0 stage 0. Because intraductal spread suspected on MRI could not be identified on ultrasonography and the extent of spread was unclear, total mastectomy was performed. The intramammary lesion was presumed to be DCIS, and no axillary lymph node enlargement was detected on imaging; therefore, SNB was omitted. Pathological examination of the erythematous area of the lower outer areola showed scattered atypical (Paget) cells containing mucus within the thickened epidermis. DCIS was identified immediately beneath the nipple, with lobular neoplasia noted deeper and laterally. No pathological lesions were identified within the nipple itself. The immunohistochemical findings of the Paget lesion and the DCIS component were identical, showing estrogen receptor positivity in 50% of tumor cells, progesterone receptor expression in <1%, and HER2 score 2+. However, despite their similar immunohistochemical profiles, no histological continuity was identified between the Paget lesion located in the lower outer areola and the intramammary DCIS located directly beneath the nipple (**Fig. 6**). The lobular neoplasia component could not be evaluated because the lesion was small and was lost during thin-sectioning.

## DISCUSSION

Paget’s disease is characterized by the proliferation of large, pale-staining atypical (Paget) cells within the epidermis and is classified into mammary and extramammary Paget’s disease. Extramammary Paget’s disease is an epithelial malignancy arising in apocrine gland–rich skin areas, such as the vulva, perianal region, and axilla, and is conceptually distinct from Paget’s disease of the breast. In contrast, Paget’s disease of the breast is regarded as a subtype of breast cancer in which malignant epithelial cells are present within the epidermis of the nipple–areolar complex and are frequently associated with an underlying intramammary carcinoma.^2)^

Several theories have been proposed regarding the pathogenesis of Paget’s disease of the breast. The epidermotropic theory suggests that malignant ductal epithelial cells migrate along the lactiferous ducts and infiltrate the epidermis of the nipple or areola. Since Jacobaeus reported in 1904 that Paget cells originate from underlying ductal carcinoma, this theory has been widely accepted.^3)^ Conversely, the intraepidermal transformation theory proposes that Paget cells arise independently within the epidermis, possibly from multipotent basal keratinocytes or mammary gland–related cells such as Toker cells.^4)^ In the present case, Paget cells were confined to the epidermis of the lower outer areola, while DCIS was identified immediately beneath the nipple. No pathological lesion was identified within the nipple epidermis itself. Although the Paget lesion and the DCIS component shared identical immunohistochemical profiles, no histological continuity between the two lesions was identified in the examined sections. Nevertheless, the possibility that microscopic continuity was not captured because of the inherent limitations of routine tissue sectioning cannot be excluded, particularly given the complex 3D anatomy of the nipple–areolar ductal system. Accordingly, direct epidermotropic spread could not be histologically demonstrated in this case and cannot be definitively excluded. At the same time, the unusual presentation limited to the areola without nipple involvement raises the possibility of an alternative pathogenic mechanism, such as intraepidermal transformation; however, this interpretation remains speculative.

Although Paget’s disease of the breast is commonly considered to involve the nipple, several cases without nipple lesions have been reported. Broecker et al. described the first such case, in which Paget cells were confined to erythematous breast skin without an underlying intramammary lesion, supporting the intraepidermal transformation hypothesis.^5)^ Van der Putte et al. reported a variant confined to the areolar skin with an intact nipple and suggested that mammary gland–related cells (Toker cells) may serve as a cellular origin in selected cases.^6)^ In Japan, among 42 reported cases of mammary Paget’s disease documented in the Ichushi-Web database between 2014 and 2024, 6 cases lacked nipple involvement. These cases exhibited heterogeneous clinical features and were frequently associated with intramammary lesions, including invasive carcinoma and DCIS (**Table 1**). These findings indicate that the absence of nipple involvement does not exclude underlying breast carcinoma and may contribute to diagnostic delay, particularly when lesions are initially treated as benign dermatologic conditions.

**Table 1:** Reports of mammary Paget’s disease without nipple involvement in the Ichushi-Web database (2014–2024)

Cases (reference)	Age (years)	Skin findings	Procedure	Mammary lesion
1^11)^	82	Invasive erythema of the areola	Bp	None
2^12)^	78	None	Bt + SNB	Apocrine carcinoma
3^13)^	50	None	Bt + SNB → Ax (11 metastases)	IDC
4^14)^	61	Mole	Bt + SNB	DCIS
5^15)^	76	Areolar erosion	Bt + Ax level I (n0/3)	IDC
6^16)^	70s	None	Bt + SNB	Mucinous carcinoma

Ax, axillary lymph node dissection; Bp, partial mastectomy; Bt, mastectomy; DCIS, ductal carcinoma in situ; IDC, invasive ductal carcinoma; SNB, sentinel lymph node biopsy

Surgical management of mammary Paget’s disease has traditionally involved mastectomy due to frequent involvement of the nipple–areolar complex. Although breast-conserving approaches, including nipple-sparing or circular partial mastectomy, have been reported,^7)^ accurate preoperative assessment of disease extent is critical for selecting an appropriate surgical strategy. In the present case, although MRI suggested limited ductal involvement near the nipple, the precise extent of disease could not be clearly delineated because of poor correlation with ultrasonographic findings and the atypical distribution of the Paget lesion within the areola. Furthermore, the possibility that nipple preservation might not be feasible was explained to the patient, and after thorough discussion, she expressed a preference for total mastectomy.

SNB was omitted because preoperative imaging demonstrated no evidence of invasive carcinoma or lymph node metastasis and the clinical stage was cTisN0. Chen et al. reported that invasive carcinoma was present in approximately 50% of cases of Paget’s disease of the breast,^4)^ and Lin et al. reported that the positivity rate of SNB in patients with Paget’s disease of the breast with intramammary lesions was 17%.^8)^ The National Comprehensive Cancer Network guidelines classify SNB for Paget’s disease of the breast as a Category 2A recommendation.^9)^ Therefore, omission of axillary staging should be carefully considered on an individual basis.

According to the revised 19th edition of the Japanese General Rules for Clinical and Pathological Recording of Breast Cancer,^10)^ Paget’s disease of the breast may be diagnosed when no contiguous noninvasive or invasive carcinoma is identified within the breast. In the present case, because histological continuity between the Paget lesion and the underlying DCIS could not be demonstrated, the diagnosis of Paget’s disease of the breast was considered appropriate under the revised criteria.

Cases presenting solely with areolar skin changes without nipple involvement are particularly prone to delayed diagnosis, as they are more likely to be initially managed by dermatologists. Awareness that mammary Paget’s disease can occur without nipple lesions is therefore essential, and prompt skin biopsy should be considered for refractory areolar lesions.

## CONCLUSIONS

It is important to keep in mind that Paget’s disease of the breast can occur without nipple lesions and that prompt skin biopsy should be considered for refractory areolar lesions to avoid diagnostic delay.

## DECLARATIONS

### Availability of data and materials

The datasets supporting the conclusions of this article are included within the article.
